# Revising the diagnosis of congenital amusia with the Montreal Battery of Evaluation of Amusia

**DOI:** 10.3389/fnhum.2015.00161

**Published:** 2015-04-01

**Authors:** Jasmin Pfeifer, Silke Hamann

**Affiliations:** ^1^Amsterdam Center for Language and Communication, University of AmsterdamAmsterdam, Netherlands; ^2^Institute for Language and Information, Heinrich-Heine-UniversityDüsseldorf, Germany

**Keywords:** Congenital amusia, MBEA, SDT, web-based testing, prevelance

## Abstract

This article presents a critical survey of the prevalent usage of the *Montreal Battery of Evaluation of Amusia* (MBEA; Peretz et al., 2003) to assess congenital amusia, a neuro-developmental disorder that has been claimed to be present in 4% of the population (Kalmus and Fry, 1980). It reviews and discusses the current usage of the MBEA in relation to cut-off scores, number of used subtests, manner of testing, and employed statistics, as these vary in the literature. Furthermore, data are presented from a large-scale experiment with 228 German undergraduate students who were assessed with the MBEA and a comprehensive questionnaire. This experiment tested the difference between scores that were obtained in a web-based study (at participants’ homes) and those obtained under laboratory conditions with a computerized version of the MBEA. In addition to traditional statistical procedures, the data were evaluated using *Signal Detection Theory* (SDT; Green and Swets, 1966), taking into consideration the individual’s ability to discriminate and their response bias. Results show that using SDT for scoring instead of proportion correct offers a bias-free and normally distributed measure of discrimination ability. It is also demonstrated that a diagnosis based on an average score leads to cases of misdiagnosis. The prevalence of congenital amusia is shown to depend highly on the statistical criterion that is applied as cut-off score and on the number of subtests that is considered for the diagnosis. In addition, three different subtypes of amusics were found in our sample. Lastly, significant differences between the web-based and the laboratory group were found, giving rise to questions about the validity of web-based experimentation.

## Introduction

Congenital amusia is a perceptual disorder that affects music and speech perception. Congenital amusics do not suffer from a hearing deficit nor do they have any form of brain lesion (Ayotte et al., 2002). Rather, the disorder is an innate one and the exact neural underpinnings are still under investigation. Therefore, no neurological markers can be used to diagnose amusia. Instead, research has revealed several behavioral markers, such as pitch perception deficits and a pitch memory deficit. The main tool used to diagnose amusia nowadays is the *Montreal Battery of Evaluation of Amusia* (MBEA; Peretz et al., 2003), which was originally developed to confirm acquired amusia in patients with brain lesions.

In the present study, we first describe the set-up of the MBEA and give an overview of its current usage and limitations. Section “Materials and Methods” presents a large-scale study that compares web-based with laboratory-based usage of the MBEA, and evaluates the MBEA scores with data on musical performance additionally obtained with a questionnaire. The results of this experiment are presented in Section “Results.” A discussion of the results is given in Section “Discussion.”

### The Montreal Battery of Evaluation of Amusia

The MBEA is a test battery developed with the main objective of assessing the musical abilities of brain-damaged patients that suffer from acquired amusia, but is nowadays used to diagnose congenital amusia. It consists of six subtests, three of which test melodic organization (scale, contour, and interval subtest), two test temporal organization (rhythm and meter subtest) and one tests melodic memory (memory subtest), based on a model of music processing summarized by Peretz and Coltheart (2003).

All six subtests use a selection of musical phrases that were specifically composed for this purpose according to the principles of the Western tonal system. These phrases are monophonic, i.e., they consist of a single voice, and they last 3.8–6.4 s (mean of 5.1 s) for all but the metric test, where they are polyphonic and twice as long (with a mean of 11 s). The procedure is the same for the first four subtests (scale, contour, interval, and rhythm): The participants are presented with two practice trials and 31 experimental trials. A trial consists of a target melody and a comparison melody (thus a stimulus pair), which are separated by a 2-s silent interval. Each trial is preceded by a warning tone and followed by a 5-s silent interval. Fifteen trials have comparison melodies that are identical to the target melody and 15 have comparison melodies that are altered in one note with respect to the target melody: In the scale subtest, the altered melodies violate the key but keep the overall contour intact; in the contour subtest, they violate the contour while keeping the key intact; and in the interval subtest, key and contour are kept intact but the pitch interval is violated. For the rhythm subtest, the rhythmic grouping of the comparison melody is changed by altering the duration of two adjacent notes. In addition to those 30 trials, each subtest contains a catch trial to ensure that the participants are paying attention and not simply guessing. For the catch trial, the pitch of the comparison melody was changed randomly, so that there is a clearly noticeable difference between the two melodies. For the first four subtests, participants are asked whether the two melodies they hear are the same or different, following an AX design. The last two subtests (meter and memory) follow a different design. In the meter subtest, 30 two-phrase sequences in duple or triple meter are used, and the participants have to judge whether the presented melody is a march or a waltz. The memory subtest presents again only single melodies, half of which already occurred in the previous subtests, the other half is new, and participants have to indicate for each melody whether they have heard it before during the previous subtests or whether it is new.

The MBEA was used by Peretz et al. (2003) to test 160 participants without known neurological problems, who were not selected for musical ability. For each participant, the number of correct responses per subtest and an average score of the six subtests was calculated. As cut-off scores for congenital amusia, Peretz et al. (2003) propose 2 SD below the mean of the 160 participants, thus an average score below 21.6, or 76.6%, cf. **Table 1**.

**Table 1:** Montreal Battery of Evaluation of Amusia (MBEA) test scores for the six subtests and average score in the study by Peretz et al. (2003, p. 66).

	Scale	Contour	Interval	Rhythm	Meter	Memory	Average
Mean correct responses	27	27	26	27	26	27	27
SD	2.3	2.2	2.4	2.1	2.9	2.3	1.6
Cut-off score (mean – 2 SD)	22	22	21	23	20	22	21.6^∗^
Cut-off in %	73.3	73.3	70	76.7	66.7	73.3	72.2^∗^
Participants with perfect score (%)	17	9	7	15	14	10	3
Participants below cut off (%)	3	1	1	1	1	1	2

^∗^Peretz et al. (2003, p. 69) list an average cut-off score of 23 and a cut-off percentage of 78%, which is probably due to a rounding error (see Wise, 2009, p. 115).

According to Peretz et al. (2003, p. 65), the MBEA subtests provide a *sensitive* measure since less than 20% of the participants obtain perfect scores for each subtest, and only 3% of the participants obtained a perfect score for all subtests (see **Table 1** row 5), while less than 2% (three participants) had average scores that were below 2 SD of the mean (**Table 1** row 6). These average scores approximate a normal distribution, though the scores for the individual subtests display a skew to the right. Peretz et al. (2003) furthermore state that the MBEA displays *test–retest* *reliability*, based on a retest of 28 participants 4 months after initial testing, though the performance of these participants improved (p. 66).

Peretz et al. (2003) *validated* the MBEA with two subtests (melody and meter) of the *Musical Aptitude Profile* (MAP; Gordon, 1965), a test battery widely used in North America to test musical abilities. These two subtests, which were chosen because they were closest in content and format to the MBEA, were administered to 68 subjects. These participants obtained similar levels of performance for the MBEA and the MAP, and the two scores positively correlated (*r* = 0.53, *p* < 0.01).

### Applications and Limitations of the MBEA

Currently most studies investigating congenital amusia utilize the MBEA, including those performed by researchers who are not associated with Peretz’ research group (Foxton et al., 2004, 2006; Patel et al., 2005, 2008; Douglas and Bilkey, 2007; Mandell et al., 2007; McDonald and Stewart, 2007; Loui et al., 2008, 2009, 2011; Nguyen et al., 2009; Tillmann et al., 2009, 2011a,b, 2012; Wise, 2009; Jiang et al., 2010, 2012a,b, 2013; Liu et al., 2010, 2012a,b, 2013; Williamson and Stewart, 2010; Williamson et al., 2010, 2011, 2012; Omigie and Stewart, 2011; Hamann et al., 2012; Loui and Schlaug, 2012; Omigie et al., 2012a,b, 2013; Thompson et al., 2012; Albouy et al., 2013a,b; Launay et al., 2014; Pfeifer and Hamann, 2014).

The actual application of the MBEA differs in terms of number of subtests, items, and cut-off scores that are employed, in their mode of testing (web-based or in the laboratory) with or without additional questionnaire, in their predictions on the prevalence of amusia, and in whether they differentiate subtypes of amusia. In the following subsections, we summarize and discuss the different usages found in the literature.

#### Scoring and Subtests

All studies testing congenital amusia calculate a score based on the sum of correct answers without distinguishing between different types of stimuli or answer categories. They usually also calculate an average score but include different numbers of subtests.

Isabelle Peretz and her colleagues use all six subtests of the MBEA (Hyde and Peretz, 2003, 2004; Peretz et al., 2005, 2007, 2008, 2009, 2012; Hyde et al., 2006, 2007, 2011; Moreau et al., 2009; Hutchins et al., 2010a,b; Nan et al., 2010; Cousineau et al., 2012; Mignault Goulet et al., 2012; Hutchins and Peretz, 2013; Moreau et al., 2013; Phillips-Silver et al., 2013) use all six subtests of the MBEA. These studies use the scores by Peretz et al. (2003) as cut-off score. In the early studies by Ayotte et al. (2002) and Peretz et al. (2002) a cut-off score of 3 SD below mean was used. As already pointed out by Wise (2009, p. 43), it is not clear why this change from 3 to 2 SD was made, but it resulted in more people being assessed as having amusia.

The research group led by Lauren Stewart and her colleagues uses only the first four subtests of the MBEA and calculates the sum of the first three, pitch-based, subtests (McDonald and Stewart, 2007; Liu et al., 2010, 2012a, 2013; Williamson and Stewart, 2010; Williamson et al., 2010, 2011, 2012; Omigie and Stewart, 2011; Omigie et al., 2012a,b, 2013; Thompson et al., 2012). As cut-off score, they use 65 out of 90 correct answers on the first three subtests (72% correct).

Several large-scale studies using all six subtests of the MBEA employ cut-off scores that are based on the means they obtain for their own participants. Cuddy et al.’s (2005) 100 control participants (subjects who reported not to be tone deaf) achieved lower mean correct responses than the control group by Peretz et al. (2003; 87% compared to 91%). As a result, Cuddy et al. (2005) set their cut-off scores at 2 SD below the mean of their controls, thus at 72%, resulting in 3–5% of the participants being diagnosed as amusic (as opposed to 18% with Peretz et al.’s (2003) cut-off scores). These scores are much lower than the ones obtained by Peretz et al. (2003), with the exception of the score for the memory test, see **Table 2** rows 1 and 2 compared to the last two rows. Wise (2009), who uses 24 test items per subtest instead of 30, also employs cut-off scores that lie 2 SD below the means of her own 24 controls (participants without self-reported problems in music perception and performance). These scores were mostly lower than the ones used by Peretz et al. (2003), cf. **Table 2** rows 3 and 4. In a study on the presence of amusia in native speakers of a tone language, Nan et al. (2010) tested 117 Mandarin speakers with no self-declared musical problems. Their cut-off scores are also given in **Table 2** (rows 5 and 6). These percentages are comparable to the ones by Wise’s control participants (though markedly lower for the meter subtest) and thus also lower than the original scores proposed by Peretz et al. (2003).

**Table 2:** MBEA cut-off scores for the six subtests and the average score by control subjects in the large-scale studies by Cuddy et al. (2005), Wise (2009), Nan et al. (2010) and Peretz et al. (2003) (for comparison).

Source		Scale	Contour	Interval	Rhythm	Meter	Memory	Average
Cuddy et al. (2005) *N* = 100	Cut-off scores	20.1	19.4	18.6	20.2	15.1	22.7	21.5
	Cut-off (%)	67.0	64.7	62.0	67.3	50.3	75.7	71.7
Wise (2009) *N* = 24	Cut-off scores	21.5	19.7	19.8	23.2	19.4	20.8	22.4
	Cut-off (%)	71.5	65.7	66.1	77.4	64.6	69.3	74.6
Nan et al. (2010) *N* = 117	Cut-off scores	19.3	20.9	17.7	22.0	16.2	21.5	21.5
	Cut-off (%)	64.2	69.6	59.0	73.3	53.9	71.8	71.7
Peretz et al. (2003) *N* = 160	Cut-off scores	22	22	21	23	20	22	21.6
	Cut-off (%)	73.3	73.3	70	76.7	66.7	73.3	72.2

Not all studies provided cut-off scores both in absolute numbers and in percentage, the missing data were calculated by the present authors.

Almost all studies use *average cut-off scores* to diagnose amusics, i.e., the performance on an individual subtest does not matter as much, especially when six subtests are used. An example for this is the study by Ayotte et al. (2002), where the average score of every amusic is 3 SD below the mean of the controls, but when considered on individual subtests, none of the 11 amusics failed all the subtests and some scored below the cut-off for only two subtests.

The practice of adding up all correct responses to calculate a score for the MBEA is criticized by Henry and McAuley (2013), as it might misdiagnose people as amusics who have a large response bias but normal discriminatory abilities. They propose the use of *Signal Detection Theory* (SDT; Green and Swets, 1966; Macmillan and Creelman, 2005), which is a psychophysical approach to measuring performance that takes into account the individual’s response bias and their ability to discriminate, both important considerations for testing a population with a perceptual deficit. Henry and McAuley (2013) compare the performance of participants who completed the standard MBEA with the performance of participants who additionally had to rate how confident they were of their answers. With these confidence scores, Henry and McAuley (2013) computed SDT scores and found a potential misclassification of 33%.

A possible *misdiagnosis* of amusics could be ascribed to a high rate in Type II error, thereby including individuals with a large response bias who have otherwise normal perceptual abilities. This would mean that by using SDT, a more rigorous standard of diagnosis would be employed, leading to fewer Type II errors. This consideration is especially important when (re-)assessing studies that have obtained null results. It seems possible that these studies included a large group of misdiagnosed individuals, thereby tainting the results.

Wise (2009) and Henry and McAuley (2010) point out the *negative skew* in the distribution of scores on the individual subtests, and furthermore that most studies using the MBEA apply parametric statistics without testing whether their data are normally distributed (exceptions are Douglas and Bilkey, 2007; Provost, 2011).

Some studies use MBEA subtests for *screening*, which could lead to potentially higher MBEA scores in the later testing; recall the improved performance by participants who were retested after 4 months in the study by Peretz et al. (2003, p. 66). Such a potential learning effect for participants screened with MBEA subtests hinders the interpretation and cross-study comparison of reported final scores.

As we could see, there is no agreement on the cut-off scores and the number of employed subtests. Both vary considerably across studies which makes cross-study comparisons difficult if not impossible. In order to employ the MBEA as diagnostic tool, a standardized usage would be necessary.

#### Web-based versus Laboratory Testing

In recent years, web-based research has become more and more common. While the MBEA is mostly conducted in a laboratory, some studies on congenital amusia employ web-based MBEA subtests for pretesting, e.g., Lauren Stewart and colleagues, who use a web-based pretest consisting of the scale and the rhythm subtest of the MBEA.

Peretz et al. (2008) proposed a *web-based amusia test* based on the MBEA. This test consists of three conditions with a total of 72 melodies based on 12 melodies from the MBEA. The task of the experiment was to spot incongruities that were inserted in these melodies and not a comparison of two melodies, as in the MBEA. In one condition, off-beat tones or silences were inserted, thereby altering the meter of the phrase. In the other two conditions, a mistuned note or an out-of-key note were inserted, respectively. Peretz et al. (2008) used the MBEA, on which this test is based, to validate it by correlating the scores on the MBEA subtests with scores in these subtests. Similar to the MBEA, the off-beat test is shown not to be normally distributed. The average score is described, but not statistically shown, to be normally distributed, while visual inspection of the provided material also reveals a skew in the data. They also mention discrepancies between the two tests: 19% of people diagnosed as amusic would not be diagnosed as such with the online test.

A discrepancy between web-based results and laboratory results has often been observed in psychological research. Krantz and Dalal (2000) comment that this does not demonstrate a lack of validity of web-based experiments, since most variables seem not to be influenced by varying environments. However, they also point out that auditory research is an exception to this observation as a stable and quiet environment is crucial for the success of this type of experiment. For the assessment of amusia with a web-based version of the MBEA, this could mean severe misdiagnoses of participants.

#### Use of Additional Questionnaires

In addition to testing with the MBEA, many studies report the usage of a *questionnaire* pertaining to information on general education, music education, language background, and musical performance such as singing and dancing (Cuddy et al., 2005; Wise, 2009; Provost, 2011). In most cases where a questionnaire was used, it is not reported how it is analyzed in relation to the MBEA results (e.g., Ayotte et al., 2002; Peretz et al., 2007; Liu et al., 2012b). One of the exceptions is Peretz et al. (2008) with 101 items on demographic and music-related information. However, only correlations between a small number of questionnaire items (age, gender, years of education, and music training) and MBEA test scores are reported.

Questionnaires could in principle provide valuable additional information in the assessment of amusics with the MBEA, but in order to evaluate their contribution more studies are required that systematically analyze the correlation between the questions used and the MBEA scores.

#### Prevalence

The MBEA is also used to estimate the *prevalence of congenital amusia* in the general population. Most amusia studies state a prevalence of 4%, referring to Kalmus and Fry (1980) (e.g. Ayotte et al., 2002; Foxton et al., 2004; Cuddy et al., 2005; Mandell et al., 2007; Peretz et al., 2007, 2009; Liu et al., 2010, 2012b; Tillmann et al., 2010; Williamson and Stewart, 2010; Omigie and Stewart, 2011; Williamson et al., 2012; Omigie et al., 2013). Kalmus and Fry (1980) introduced the *Distorted Tunes Test* (DTT), consisting of 26 well-known tunes to assess congenital amusia (or tone deafness as they called it). Incorrect notes were inserted into 17 of these tunes. Kalmus and Fry’s (1980) criterion for the presence of amusia was the inability to detect wrong notes in at least three out of the 17 incorrect tunes. They tested 604 adults and based on this data they estimated a prevalence of 4.2%.

Recently, Peretz (2013) stated 2.5% as the prevalence of amusia in the general population, and added that the use of only the MBEA scale subtest by Provost (2011) resulted in a prevalence of 3.2%. Provost (2011) used the online study based on Peretz et al. (2008) described in Section “Web-based versus Laboratory Testing.” For the 1100 participants who completed the test and fitted the age and education criteria, scores were considered individually and any participant falling below the cut-off score on one of the three subtests was considered amusic. This yielded a total of 11.6% amusics, supporting the observation above that on-line testing yields a higher prevalence of amusia.

Henry and McAuley (2010, p. 414) point out that the MBEA, just like other methods to assess the prevalence of disorders (including dyslexia and dyscalculia), suffers from an *arbitrary cut-off* problem and that a cut-off of 2 SD from the mean in normally distributed values (as claimed for the average score of the MBEA) would by definition result in a 2.28% expected occurrence rate. The same criticism can be applied to the prevalence proposed by Kalmus and Fry (1980), as their DTT shows the same arbitrary cut-off score and lack of well-established psychometric properties (Ayotte et al., 2002; Hyde and Peretz, 2004; Henry and McAuley, 2010). Henry and McAuley (2010) therefore propose to include structured interviews with participants for predictions on prevalence (see Use of Additional Questionnaires above).

#### Subtypes of Amusia

Wise (2009) and Henry and McAuley (2010) criticize the widespread use of average scores for the MBEA, because this practice ignores heterogeneous behavior of participants across the six subtests. In the study by Ayotte et al. (2002) for instance, only the scale subtest was failed by all congenital amusics (Wise, 2009, p. 43). Wise further reports that for the rhythm and the meter subtest, half of the participants usually pass and more than half pass the memory subtest. At the same time, participants have been reported who only have problems with the rhythm subtest (Peretz et al., 2003). All this points to the existence of several subtypes of congenital amusia with a possible dissociation between pitch- and rhythm-related deficits, as already suggested by Peretz et al. (2003, p. 70). Some studies using the MBEA introduce the amusic subtype of *beat* *deafness* (Phillips-Silver et al., 2011) or *dysrhythmia* (Launay et al., 2014). Phillips-Silver et al. (2011) report a single case of rhythmic deficits with intact pitch perception, while Launay et al. (2014) identify three such cases. The opposite, intact rhythmic perception with impaired pitch perception, has also been reported by Phillips-Silver et al. (2013). Reports of a subtype with rhythm deficits are less frequent, possibly due to a low proportion of rhythm-related subtests in the MBEA. Provost (2011) proposes four different subtypes: pitch-deaf amusics, pitch-perception amusics, pitch-memory amusics, and beat-deaf amusics. The latter classification does not include an amusia type that has both a pitch perception and a rhythm perception deficit and rather focuses on different pitch abilities.

This overview shows that even though there is large overlap in the proposed subtypes of amusia, clear-cut definitions for such subtypes are still missing. Furthermore, we can conclude that it is advisable to use cut-off scores of single subtests instead of average scores in order to advance further research on subtypes of amusia.

## Materials and Methods

### Participants

Two hundred and eighty first year undergraduate students in general linguistics at the Heinrich Heine University Düsseldorf participated in our study. The participants were not preselected for the presence or absence of musical disorders such as amusia, or specific levels of musical experience. All participants gave informed written consent to participate in this study and received course credit for their participation. All data were collected in accordance with the declaration of Helsinki. The participants took a hearing test and answered a detailed questionnaire about their linguistic and musical background (experience with and attitude to music and dance, in performance and perception). An intelligence test was not performed as all participants were university students and expected to have an average to high level of intelligence.

A total of 52 students were excluded from data analysis. Eight did not have normal hearing as assessed by pure tone audiometry at 250–8000 Hz. Normal hearing was defined as a mean hearing level of 20 dB or less in both ears. Forty-five participants had a different native language than German. In order to keep the variance between participants as little as possible, these participants were excluded as well. Of the remaining 228 participants, 117 completed a web-based version of the MBEA at home and 111 a computer-implemented version in a sound-attenuated booth in our laboratory. Participant details can be found in **Table 3**. The last row in this Table shows that the two participant groups did not differ significantly in their characteristics.

**Table 3:** Descriptive statistics and results of *t-*tests comparing laboratory and web-based participant characteristics.

Group		Age	Years of education	Years of music education	Handedness	Gender
Laboratory	Mean	22.7	14.4	5.9	101 right 7 left 3 ambidextrous	90 female 21 male
	Range	20–35	12–23	0–12
Web-based	Mean	22.0	14.7	6.3	107 right 9 left 1 ambidextrous	99 female 18 male
	Range	19–36	12–22	0–17
Total	Mean	22.3	14.6	6.1	207 right 16 left 4 ambidextrous	188 female 39 male
*t*-test means	*t*	1.82	–1.06	–0.82	–	–
	*p*	0.71	0.29	0.41	–	–

*t*, test statistic of the independent samples *t*-test; *p*, probability value.

### Procedure

All participants completed the MBEA. Half of them completed a computer-implemented version in a sound-attenuated booth, where the stimuli were presented over AKG K 601 headphones using Praat (Boersma and Weenink, 2011) on a Windows XP computer. These participants could adjust the volume to a comfortable level and had unlimited time. The other half completed a web-based version of the MBEA. This group was informed before the experiment that they should use headphones and take the test in a quiet environment without any distractions. The on-screen instructions for both groups were identical. For the first four and the sixth subtest, participants received two examples with feedback before the beginning of each subtest. For the fifth (meter) subtest, participants received four examples, instructing them what a march and a waltz sound like. A detailed description of the MBEA stimuli and the general procedure was given in Section “The Montreal Battery of Evaluation of Amusia.”

The laboratory group took part in the MBEA, filled in the questionnaire and took the hearing test in the same session, which lasted about 70 min. The web-based group completed the MBEA online at home. At a later point, these participants came to the laboratory for the hearing test and to answer the questionnaire. A test administrator was always present to answer clarification questions about the questionnaire. At the end of the session, participants were allowed to ask questions about the nature of the study and a couple of weeks later they were informed about the group results.

### Scoring

The MBEA uses a same-different paradigm for the first four subtests. In such a test design, participants respond to two different types of trials (stimulus pairs) in two different ways: A stimulus pair where the comparison melody differs from the target melody is considered a hit when it is correctly identified, and a miss when it is not correctly identified. A stimulus pair with two melodies that are the same is considered a correct rejection (CR) when it is identified as identical, and scored a false alarm (FA) when it is incorrectly identified as different; see the overview in **Table 4**.

**Table 4:** Overview of stimulus types and possible responses.

Stimulus pair	Response	
	Different	Same
Different	Hit (H)	Miss (M)
Same	False alarm (FA)	Correct rejection (CR)

Following Henry and McAuley (2013), these scores were also applied to the metric subtest, where trials with a march were treated as different and trials with a waltz as same stimulus pairs, and to the memory subtest, where trials with already used melodies were treated as different and those with new melodies as same stimulus pairs.

Poor performance on the MBEA can occur for different reasons: It can be caused by a high number of FAs, a high number of misses or a combination of both. We therefore performed not only a conventional analysis of the MBEA by calculating the sum of correct responses, but also employed the SDT measures *d*^′^, as a measure of sensitivity, and *criterion location* (*c*), as a measure of participants’ response bias. Both rely on hit rate (HR) and false alarm rate (FAR). *d*^′^ is equally dependent on H and FA and allows for the fact that sensitivity should increase when H increases and decrease when FA increases. It is calculated by subtracting the inverse of the normal distribution functions of FA from H, converting them into a SD unit (*z*-scores), cf. (1), and thereby making the measure comparable across tasks (Macmillan and Creelman, 2005). A *d*^′^ score of 0 means a participant is unable to discriminate between stimuli, and the higher the *d*^′^ score (and thus the sensitivity), the better the participant discriminates between stimuli.

(1) *d*^′^ = z(HR) – z(FAR)
(2) *Criterion Location*: *c* = –0.5⋅(z(HR) + z(FAR))

The second measure, *c*, is the participants’ response bias, i.e., the tendency to favor one of the two possible responses (Macmillan and Creelman, 2005) and is calculated as in (2). Positive *c* values correspond to a tendency to respond ‘same’ and negative values correspond to a tendency to respond ‘different.’

## Results

Several analyses were performed on the data. First, the results of the web-based group and the group tested in the laboratory were analyzed and compared. In Section “Prevalence,” the cut-off scores of our sample and the prevalence that we found are compared to the cut-off scores by Peretz et al. (2003). Section “Subtests” discusses the use of combined subtests for our data. Section “Scoring with Signal Detection Theory” shows the result if SDT was applied and discusses the differences in prevalences. Resulting subgroups of amusia are discussed in Section “Subtypes.” This is followed by an analysis of the questionnaire items in relation to the MBEA scores in Section “Questionnaire.”

### Web-based versus Laboratory Testing

The web-base tested group and the group tested in the laboratory were analyzed separately, by computing the sum of correct responses, cf. **Table 5**.

**Table 5:** Sum of correct responses for the web-based and laboratory groups (absolute numbers, with maximum of 30 per subtest).

Group		Scale	Contour	Interval	Rhythm	Meter	Memory	Average
Web-based	Mean	24.97	23.86	23.21	24.87	24.09	26.34	24.56
	SD	3.03	3.38	3.89	3.80	5.29	3.09	3.94
Laboratory	Mean	24.95	24.68	24.32	25.84	26.07	27.51	25.56
	SD	2.73	3.01	3.29	2.64	3.65	1.77	3.09

**FIGURE 1:**
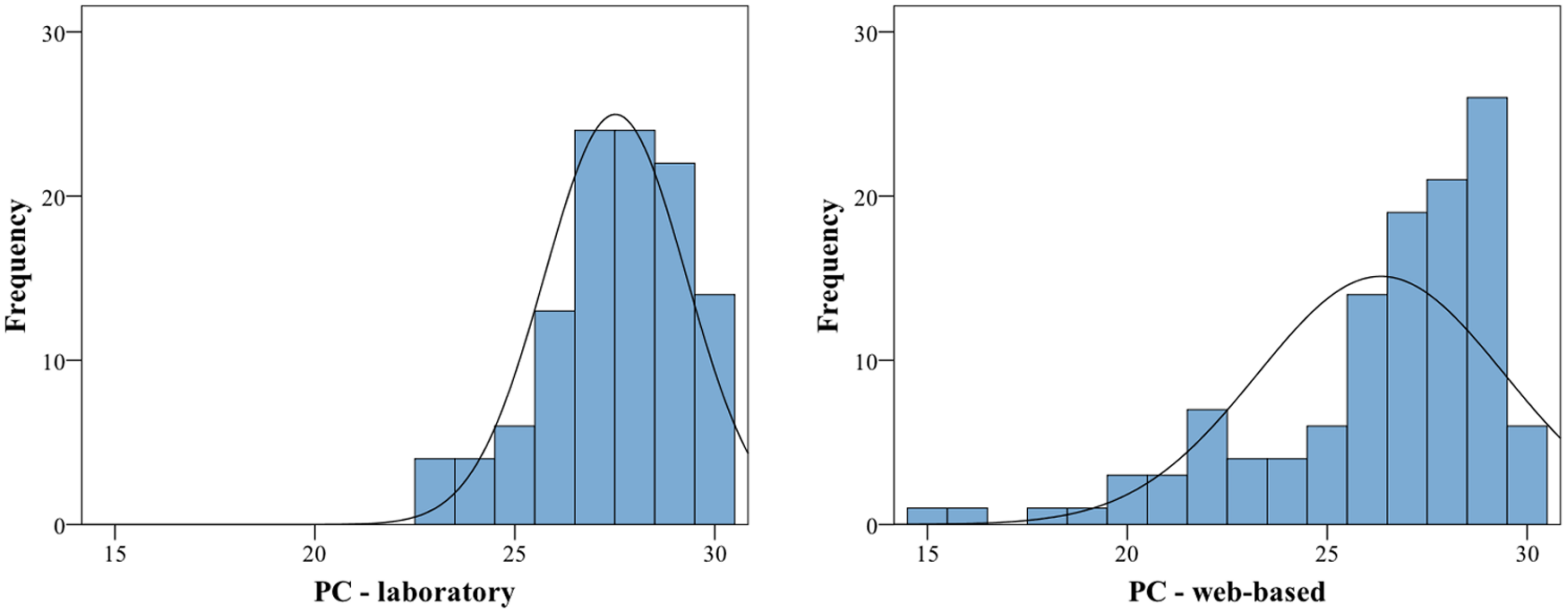
**Histograms of proportion correct for the Memory subtest for laboratory **(left)** and web-based group **(right)**.** Both exhibit a significant negative skew (the web-based group additionally exhibits a significant kurtosis), thus are deviating from a normal distribution. For statistics, cf. ****Table 6****.

Visual inspection of histograms indicated that the data for the individual subtests and for the average of all subtests are not normally distributed, for an example illustration see **Figure 1**.

The calculation of skew and Kolmogorov–Smirnov tests yielded significant results as well (for exact values see **Table 6**). All subtest scores and the average scores exhibit a negative skew and are visibly shifted toward the right. In addition, the variances between the groups are significantly different for four of the six subtests as revealed by Levene’s test and therefore the assumption of homogeneity of variance is also violated for these four tests (for exact values see **Table 6**). For these reasons, additional non-parametric tests were performed. Mann–Whitney-*U* tests revealed significant differences between the web-based and the laboratory group in four out of six subtests. The contour and interval subtest and the average of all subtests reached significance at *p* < 0.05 and the meter and memory subtests reached significance at *p* < 0.01 (for values see **Table 6**). Due to the significant differences between the web-based and the laboratory group, the data from the two groups were not collapsed, and only the data from the group tested under laboratory conditions were further analyzed.

**Table 6:** The results of variance and normality analyses, comparison between laboratory and web-based group per MBEA subtest.

Subtest	Group	Skew	SE Skew	*z* Skew	Kurtosis	SE Kurtosis	*z* Kurtosis	Kolmogorov–Smirnov Test		Levene’s Test		Mann-Whitney-Test (1-tailed)		
								*D*	*p*	*F* (10.226)	*p*	*U*	*z*	*p*
Scale	Lab	–1.06	0.23	**–4.62**	1.99	0.46	***4.37***	0.14	***0.00***	1.46	**0.23**	6239	–0.52	0.30
	Web	–0.93	0.22	**–4.18**	0.61	0.44	1.36	0.16	***0.00***					
Contour	Lab	–0.70	0.23	**–3.02**	0.05	0.46	0.12	0.12	***0.00***	2.56	**0.11**	5588	–1.83	*0.03*
	Web	–0.45	0.22	–*1.99*	–0.43	0.44	–0.96	0.12	***0.00***					
Interval	Lab	–0.81	0.23	**–3.54**	0.55	0.46	1.20	0.14	***0.00***	4.10	*0.04*	5398	–2.21	*0.01*
	Web	–0.47	0.22	–*2.09*	–0.40	0.44	–0.90	0.09	*0.01*					
Rhythm	Lab	–0.63	0.23	–*2.74*	–0.06	0.46	–0.14	0.14	***0.00***	8.86	*0.003*	5786	–1.43	0.08
	Web	–0.96	0.22	**–4.31**	0.28	0.44	0.64	0.15	***0.00***					
Meter	Lab	–1.20	0.23	**–5.23**	1.22	0.46	*2.69*	0.17	***0.00***	19.76	***0.00***	5251	–2.51	*0.01*
	Web	–0.85	0.22	**–*3.80***	0.13	0.44	0.29	0.15	***0.00***					
Memory	Lab	–0.66	0.23	–*2.86*	0.05	0.46	0.11	0.15	***0.00***	19.85	***0.00***	5309	–2.41	*0.01*
	Web	–1.39	0.22	**–6.22**	1.78	0.44	***4.01***	0.20	***0.00***					
Average	Lab	–0.69	0.23	**–3.02**	0.55	0.46	1.20	0.33	***0.00***	8.60	*0.004*	5353	–2.29	*0.01*
	Web	–0.94	0.22	**–4.18**	0.62	0.44	1.39	0.28	***0.00***					

Bold indicates *p* < 0.001, italics *p* < 0.05.

### Prevalence

The means of the sum of correct responses, SD, and different cut-off scores are given in **Table 7**. The *average* values are calculated by averaging the scores of all participants across all subtests, it is not an average of the means or SD. The *pitch average* is an average of the scores for the scale, contour, and interval subtest.

**Table 7:** Sum of correct responses for the group tested in the laboratory.

Sum of correct responses	Scale	Contour	Interval	Rhythm	Meter	Memory	Average	Pitch Average
Mean	24.95	24.68	24.32	25.84	26.07	27.51	25.48	24.64
SD	2.73	3.01	3.29	2.64	3.65	1.77	2.06	2.47
Cut-off (2 SD)	19.49	18.66	17.74	20.56	18.77	23.97	21.36	19.7
Cut-off (%)	65.0	62.2	59.1	68.5	62.6	79.9	71.2	65.7
% below cut off	4.5	7.2	7.2	4.5	6.3	7.2	5.4	6.3
Cut-off % Peretz	73.3	73.3	70	76.7	66.7	73.3	76.6	72.2
% below cut-off Peretz	14.4	22.5	15.3	20.7	7.2	0	9.01	13.5

Cut-off scores and resulting percentage of amusics based on our mean compared to Peretz et al.’s (2003) means.

Based on the average of all subtests, 5.4% of the laboratory-tested participants would be diagnosed as amusics because their scores fall below a cut-off score of 71.2% (our mean – 2 SD). If the cut-off score by Peretz et al. (2003) were applied, 9% of our sample would be categorized as amusic. However, the prevalence is different when considering the subtests individually: If only individuals who fell below the cut-off score on every subtest are considered amusic, then the prevalence with the cut-off scores based on our data sinks to 0, and with Peretz et al.’s (2003) cut-off scores it sinks to 4.5%.

### Subtests

We were further interested in an average score for all three pitch-based subtests, as this is often used in the literature. In our sample, the average cut-off score of the pitch-based subtests is 65.7%, yielding 6.3%, (in absolute numbers 7) amusics, while Peretz et al.’s (2003) cut-off scores give a prevalence of 13.5%. We also investigated how many subtests contributed to the pitch average score per subject: Of the seven amusics below our cut-off score, one fell below the individual cut-off scores on all three subtests, four fell below on two subtests and two fell below the cut-off score on only one subtest. We then considered again all participants that failed at least one of the three pitch-based subtests, i.e., not only the pitch average, which yielded a total of 13.5% or 15 individuals. It is to be noted that these are not the same 15 individuals that are categorized as amusic when using Peretz et al.’s (2003) pitch average cut-off score. The same analysis based on Peretz et al.’s (2003) cut-off scores yielded 26.7% who fell below the individual cut-off scores on all three subtests; 60% fell below on two subtests and 13.3% fell below the cut-off score on only one subtest. Again, when considering all individuals who fell below the cut-off score on at least one of the three pitch-based subtests, 35% (39 individuals) appear to be affected. It is to be noted, however, that a correlation analysis of the scores of the different subtests yielded no statistical reason to use an average score of the three pitch based subtests. Their scores correlated just as highly with the temporal subtests and the memory subtest as with each other. The scores on the contour subtest, for example, are highly significantly positively related to the scores on all other subtests (contour and scale. τ = 0.233, *p* < 0.001; and interval τ = 0.443, *p* < 0.001; and rhythm τ = 0.273, *p* < 0.001; and meter τ = 0.249, *p* < 0.001; and memory τ = 0.199, *p* < 0.001). A pitch average score can therefore only be motivated by the same component that is supposed to be tested by all three pitch-based subtests but not by a correlation between the scores on these subtests.

### Scoring with SDT

**FIGURE 2:**
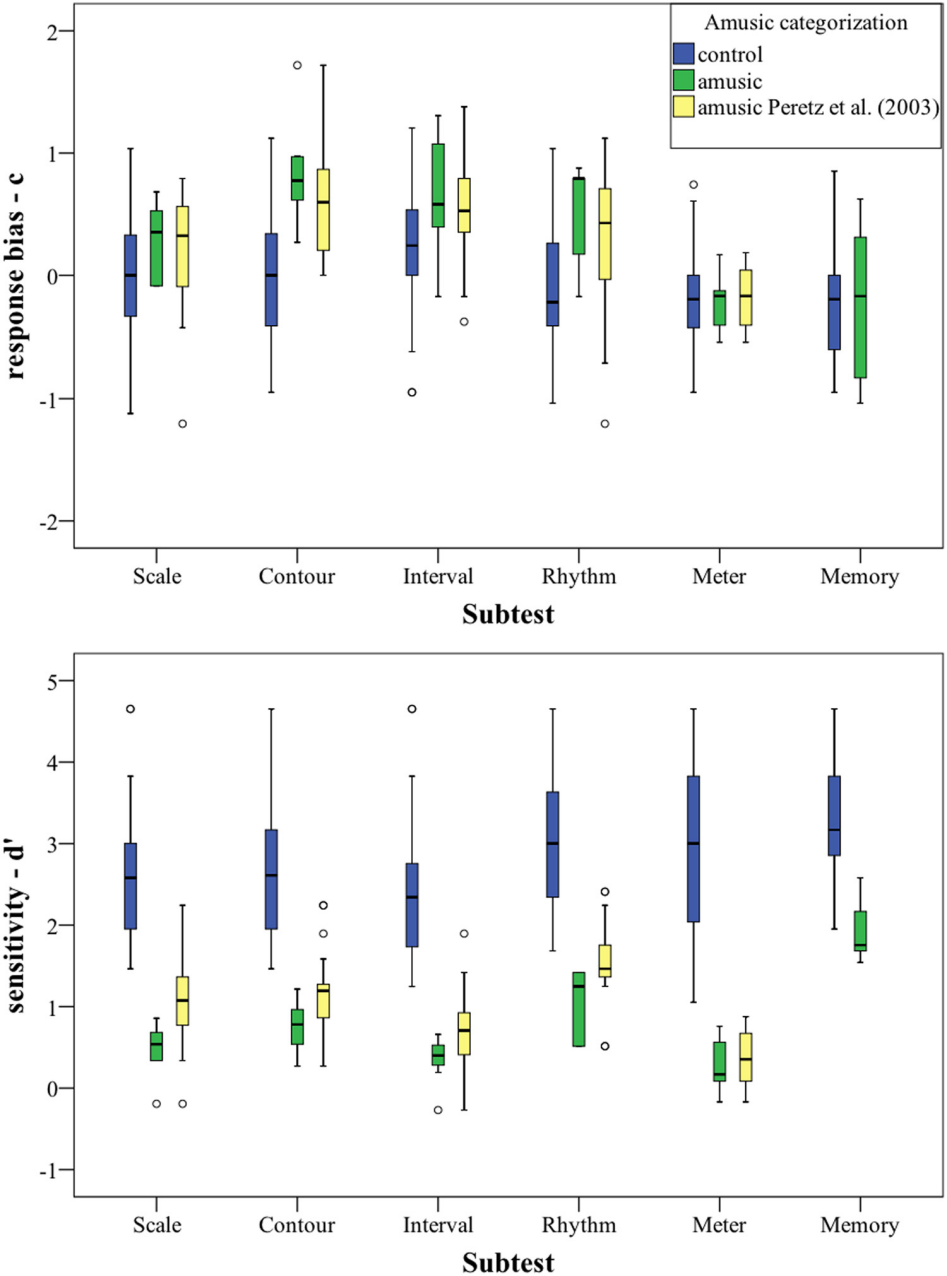
**Signal Detection Theory (SDT) scores (*d*^′^ and *c*) plotted per subtest.** Categorization based on PC-scores. For statistics, cf. ****Table 8****.

A further analysis was carried out using SDT, in order to inspect whether the obtained scores are tainted by response bias. Therefore, the means and SD of *d*^′^ and *c* were calculated for every subtest, cf. **Table 8** and **Figure 2**. An analysis of skew and kurtosis of *d*^′^ showed that the scores on all subtests are normally distributed.

**Table 8:** Means and SD of *d*^′^ and *c* for the group tested in the laboratory, including cut-off scores and percentage of amusics and controls categorization based on PC and *z*-scores used for normality analysis.

		Scale	Contour	Interval	Rhythm	Meter	Memory	Average	Pitch Average
*c*	Mean	0.01	0.15	0.29	–0.05	–0.22	–0.21	–0.00	0.15
	SD	0.55	0.54	0.48	0.55	0.34	0.43	0.29	0.46
*d*^′^	Mean	2.33	2.25	2.16	2.65	2.81	3.23	2.25	2.25
	SD	0.84	0.90	0.95	0.90	1.28	0.81	0.66	0.73
	*z* skew	–0.51	0.69	0.28	–0.19	–0.99	0.32	1.56	–0.01
	*z* kurtosis	1.28	–1.30	0.10	–1.64	–1.70	–1.15	0.61	0.15
% below cut-off score: Mean–1 SD		15.32	18.02	13.51	25.23	18.92	17.12	14.41	14.41
% overdignosis: amean–1 SD		1.80	4.50	1.80	2.70	0.00	1.80	2.70	1.80
% underdiagnosis: Mean–1 SD		2.70	0.00	0.00	7.21	11.71	11.71	1.80	2.70

The previous categorization based on our cut-off scores was kept for this analysis: The group which scored below our cut-off score was labeled “amusic,” while the group that scored below Peretz et al.’s (2003) cut-off scores (cf. **Table 7**) was labeled “amusic Peretz et al. (2003).” Our amusic group was a subgroup of the amusic group with Peretz et al. (2003) cut-off score for all of the subtests except for the memory subtest, where our cut-off score was higher, cf. **Table 7**. The rest of the participants were labeled “controls.”

In the upper part of **Figure 2**, the response bias *c* is plotted per group for every subtest. Overall, the controls groups’ *c* is located around 0, indicating that location of the border in the decision space of the controls is between the two stimulus categories. The two amusic groups have a slightly more positive bias (i.e., a tendency to answer “same”).

The lower part of **Figure 2** shows the sensitivity measure *d*^′^, the groups’ ability to discriminate, for each subtest. As can be seen, there is no overlap between our amusic group and the control group for the first five subtests, showing a clear distinction in discriminatory ability between the groups. The *d*^′^ values for the amusic group(s) are much lower than that for the controls for these five subtests, indicating that amusics have difficulties discriminating between the stimuli.

New cut-off scores based on the discriminatory ability of the groups were calculated. The cut-off score was set to be mean – 1 SD (chosen *a priori*). It is to be noted that it is an arbitrary statistical criterion. Even though the categorization varies based on the statistical criterion that is applied, this might offer a more reliable measure than averaging the sum of correct responses as the bias is factored out and participants can be categorized solely based on their ability to discriminate. The new cut-off scores and prevalences can be found in **Table 8**.

This new categorization based on discriminatory ability shows cases of over- and underdiagnosis in comparison to the previous scores. An underdiagnosis (previously categorized as control, but low discriminatory ability) does not happen for two of the three pitch-based subtests. For the scale subtest, it happens for 2.7% of all diagnosis. For the temporal subtests and the memory subtests, however, 7.2–11.2% of all participants with a low discriminatory ability are not diagnosed as amusic. Depending on the subtest, an overdiagnosis (diagnosed as amusic, but normal discrimination ability with a high bias) seems to happen in 1.8–4.5% of all diagnosis, based on the entire group. But when only considering the amusia-diagnoses based on the previous scores, then the percentage of overdiagnosis rises to 12–20% depending on the subtest, or even to 30%, when the diagnosis was based on the average score.

### Subtypes

We were also interested in different subgroups, i.e., subtypes of congenital amusia, therefore we considered the different patterns that participants exhibit on the different subtests. 53.2% scored below a cut-off score on at least one subtest, 28.8% on at least two, and 13.5% on three or more subtests. For the latter two groups, we analyzed the different subtypes. As **Table 9** shows, there are three distinct subgroups: One bigger group with below cut-off-scores on pitch and rhythm (and partly also memory) subtests and two smaller groups with low scores on only pitch and memory or only rhythm and memory subtests. As many studies also consider the average of all subtests and the pitch-average, we also calculated these. Of the 28.8%, 34.4% also had a below cut-off score on the average of all subtests and on 43.8% also scored below cut-off on the pitch average. For the population with below cut-off scores on at least three subtests, 66.6% had a below cut-off score on the average score and 80% on the pitch average score.

**Table 9:** Percentage of participants scoring below a cut-off score of (mean–1 SD) on at least two subtests.

	Two or more subtests		Three or more subtests	
	Total (%)	Below cut-off score (%)	Total (%)	Below cut-off score (%)
Total	28.8		13.5	
Only pitch	5.4 (3.6)	18.6 (15.6)	2.7 (2.7)	20.0 (20.0)
Only rhythm	9.0 (4.5)	31.3 (12.5)	0.9 (0.9)	6.6 (6.6)
Pitch and rhythm	14.4 (5.4)	50.0 (18.6)	9.9 (5.4)	73.3 (40)

The value in brackets indicates how many percent also included a below cut-off score on the memory subtest.

### Questionnaire

Our questionnaire contained 27 items: six demographic items (age, gender, education, handedness, occupation, native language(s)), 20 self-rate items about music education, attitude toward music, music habits and dancing, and one free text question (why people considered themselves unmusical, if they indicated so in the previous question). The questionnaires of the web-based and the laboratory-based group were analyzed together as they were collected under the same circumstances in the laboratory. A principal component analysis (PCA) with an oblique rotation (promax) allowed for the collapsing of the items into six factors. The PCA included 19 out of the 20 self-rate items, as the remaining item (Perception 5 – Evaluation of own perception) failed to reach the acceptable limit of 0.5 on the Kaiser–Meyer–Olkin (KMO) measure of sampling adequacy. This item was included in a first analysis but excluded from the final analysis. It is to be noted that not every participant answered every question and cases were therefore excluded pairwise. The KMO measure verified the sampling adequacy for the analysis, KMO = 0.8, and all KMO values for individual items were >0.5. Bartlett’s test of sphericity χ^2^ (171) = 1530.778, *p* < 0.001, indicated that correlations between items were sufficiently large for PCA. Six components had eigenvalues over Kaiser’s criterion of 1 and in combination explained 67.65% of the variance. **Table 10** shows the factor loadings after rotation. The items that cluster on the same components suggest that component 1 represents *perception* but also contains clapping, component 2 *music education*, component 3 *dancing*, component 4 *singing/production,* component 5 *self-assessment of musicality*, and component 6* music listening habits*. These components were then entered into a multiple regression analysis in order to calculate their influence on the MBEA-scores.

**Table 10:** Summary of principal component analysis (PCA): Rotated pattern matrix with factor loadings, ordered according to the factor loadings per component.

Questionnaire item	Component					
	1	2	3	4	5	6
Perception 1 – melodies without lyrics	**0.85**	–016	–0.19	–0.04	–0.07	0.10
Perception 3 – piano tones	**0.84**	–0.00	–0.08	–0.01	0.02	–0.02
Perception 2 – off/wrong singing	**0.72**	0.01	–0.02	0.15	0.07	–0.06
Clapping	**0.69**	0.13	0.20	–0.22	–0.09	0.17
Singing 3 – notice wrong singing and correct it	**0.52**	–0.07	0.10	0.37	0.06	–0.08
Perception 4 – surrounded by music as child	**0.44**	0.19	0.12	0.19	–0.03	–0.12

Music education 2 – age of onset	0.01	**0.90**	–0.08	–0.05	–0.09	–0.02
Music education 4 – frustration	0.00	**0.86**	0.00	–0.04	0.00	0.03
Music education 3 – years of lessons	–0.14	**0.82**	0.09	–0.03	–0.09	0.06
Music education 1 – type of education	0.15	**0.60**	0.00	0.06	0.02	–0.12
Music education 5 – still playing/singing	–0.08	**0.52**	–0.16	0.19	0.32	0.05

Dancing 2 – quality – own assessment	0.03	–0.01	**0.95**	–0.06	0.00	–0.05
Dancing 1 – quantity/frequency	–0.14	–0.03	**0.91**	0.13	0.04	0.04

Singing 1 – when alone	–0.08	–0.01	0.09	**0.85**	–0.04	0.13
Singing 2 – in public	0.13	0.01	–0.05	**0.80**	–0.02	–0.01

Unmusicality 1 – family members	–0.15	–0.11	–0.01	0.04	**0.93**	–0.01
Unmusicality 2 – qwn assessment	0.17	0.04	0.08	–0.13	**0.83**	0.05

Listening habits 1 – quantity listening to music	–0.02	–0.02	–0.03	0.27	–0.13	**0.80**
Listening habits 2 – attitude toward music	0.09	0.02	0.02	–0.12	0.17	**0.77**

Bold indicates that this item loads onto the component given in the first line.

The multiple regressions was performed separately for every subtest. The regression analysis again included only the MBEA-scores obtained in the laboratory-based group and only those participants who answered all questionnaire items, so as not to include participants with missing values. 76 participants remained in this analysis step. The six components were entered as predictors and *d*^′^ was used as outcome variable. To use *d^′^*-scores in this context has an advantage over using PC scores, as these were shown not be normally distributed, which is one of the assumptions that has to be met for a regression analysis. The assumptions of multicollinearity and independent errors were true (cf. **Table 11** collinearity statistics and Durbin–Watson test, respectively). The assumptions of linearity and homoscedasticity were visually inspected and also true.

**Table 11:** Summary of multiple regression analysis predicting MBEA scores per subtest from components.

		Coefficients		Collinearity statistics		Model fit		
Subtest	Components included	*B*	β	VIF	Tolerance	*R*^2^	Durbin–Watson	ANOVA *F*-ratio
Scale	Constant 1 perception 2 music education	***2.33*** *0.20* *0.27*	0.23 0.33	0.83 0.83	1.20 1.20	***0.22***	1.87	***10.23***

Contour	Constant 1 perception 2 music education	***2.20*** ***0.29*** *0.21*	0.31 0.24	0.83 0.83	1.20 1.20	***0.22***	2.41	***10.12***

Interval	Constant 2 music education	***2.01*** **0.46**	0.50	1.00	1.00	***0.25***	1.93	***24.70***

Rhythm	Constant 2 music education	***2.75*** *0.24*	0.29	1.00	1.00	*0.09*	2.07	*6.90*

Meter	Constant 2 music education 6 attitude	***2.76*** ***0.73*** *0.25*	0.56 0.21	0.99 0.99	1.01 1.01	***0.38***	2.13	***22.30***

Memory	Constant 5 own musicality assessment	***2.93*** *0.31*	0.33	1.00	1.00	*0.11*	2.10	*8.97*

Average	Constant 1 perception 2 music education	***2.22*** *0.21* ***0.30***	0.30 0.44	0.83 0.83	1.20 1.20	***0.39***	2.36	***23.30***

Bold and italics indicates *p* < 0.001, italics *p* < 0.05.

Different models were fit to the data, excluding non-significant predictors, until the best fitting regression model was found for every subtest. **Table 11** summarizes these models.

*R*^2^ can be used as a measure of how much variability of the outcome variable is accounted for by the predictors. Twenty-two percent to 25% of the variation in MBEA scores of the three pitch-based subtest can be predicted by the first and second component. Only 9% of the variation of the rhythm scores can be explained by questionnaire items, while 38% can be explained for the meter subtest, based on component 2 music education and component 6 listening habits/attitude. Eleven percent of the variability in memory scores can also be explained by questionnaire items, more specifically by one’s own musicality assessment. Lastly, the 39% of the variation in the average score can be predicted by component 1 and 2, perception and music education, respectively. The standardized beta value (in SD units) is used as a measure of how much the outcome variable is changed by a change of the predictor. The standardized beta value on the interval subtest (standardized β = 0.50), for example, indicates that if the score of component 2 increases by 1 SD (1.02), the *d^′^* score increases by 0.50 SD (0.47).

## Discussion

In the present study, a comparison of the MBEA scores for our laboratory-tested participants calculated both on the basis of the sum of correct answers and the Signal Detection measure *d^′^* (with mean – 1SD as cut-off) yielded different diagnoses. With the PC-based measure, 12–20% of amusia diagnoses are misdiagnoses of people who could be shown to have a normal discriminatory ability but simply a larger response bias. If we consider the average score across all subtests, then this misdiagnosis rises to 30%. This number confirms Henry and McAuley’s (2013) finding of a PC-based misdiagnosis of 33%. At the same time, the PC-based scores in our study fail to diagnose 7.2–11.2% of the participants with a low discriminatory ability as amusic.

Furthermore, we found that 28.8% of the laboratory-tested participants scored below cut-off score on two or more subtests and of those only 34.4% also scored below cut-off on the average of all subtests. This shows that a substantial number of participants with an impaired discriminatory ability is missed by using average scores for the diagnosis of congenital amusia. In addition, we could show that for the average pitch score, which is often employed in MBEA studies (see the overview in Section “Scoring and Subtests”), the scores on the pitch subtests correlated as highly with each other as with the other subtests, giving no statistical reason for using an average pitch score.

The misdiagnosis of congenital amusia has implications for the inclusion of participants in scientific studies and therefore the expansion of knowledge about congenital amusia. At the same time, the diagnosis has personal consequences for the individual in question, just as in the case of acquired amusia. Many possible amusics who come to our lab actively seek answers as to why their perception seems to be different from that of other people. These participants deserve an accurate assessment of their abilities. Using *d*^′^ to assess amusics’ discriminatory ability reflects their abilities more accurately than using the sum of correct answers.

We were furthermore also interested whether our data provided evidence for different subtypes of amusia, as have been proposed in previous studies (see the discussion in Section “Subtypes of Amusia”). For our group of participants that performed below the cut-off score of mean – 1 SD and failed at least two subtests, we found three subgroups: A group that only exhibits pitch deficits (18.6% of amusics), one with only rhythmic deficits (31.4% amusics) and another with pitch and rhythm deficits (50% of amusics). All of these groups contained participants with and without low performance on the memory subtest. When considering only participants who failed at least three subtests, then the same three groups remain. However, only 6.6% of amusics exhibit a rhythm deficit, 20% exhibit a pitch perception deficit, and 73.3% exhibit both. The probability of these three types is the same as if the three failed tests were randomly distributed across all six subtests. The high co-occurrence of pitch and rhythm deficits could be due to the very high correlations between the various subtests, which were found above. The percentage of the rhythm subtype sinks so drastically due to the imbalance of pitch and rhythm tests on the MBEA. In order to score below cut-off on three subtests with only one rhythm perception deficit is impossible; therefore also a memory deficit has to be present. Reports of subtypes with pitch deficits or pitch and rhythm deficits are more frequent than cases with rhythmic problems only (Phillips-Silver et al., 2011 – 1 case; Launay et al., 2014 – 3 cases), possibly due to the low proportion of rhythm-related subtests in the MBEA. We therefore propose that additional tests assessing rhythmic abilities, e.g., a part of the Beat Alignment Test by Iversen and Patel (2008), should be considered as a supplement to the MBEA. This might make a further differentiation of subtypes of congenital amusia and a clearer definition of them more feasible in the future. This finding again also supports our view that an average score should not be used for the diagnosis of amusia [see also Wise (2009) and Henry and McAuley (2010)], as it does not reflect the heterogeneous behavior of participants across the six subtests. The evaluation of scores on individual subtests, on the other hand, can lead to misdiagnosis of people as amusic who simply did not pay enough attention to the experiment. Though we tried to filter out such participants by the so-called catch trials, these catch trials can be detected without focused attention and therefore might not be an adequate way of controlling for such possible false positive diagnoses.

In addition to the MBEA scores, we also analyzed the information from our questionnaire.

The questionnaire contained 27 items, which were reduced by PCA to six components. These encompassed perception, music education, dancing, singing/production, listening habits, and self-assessment of musicality. Three of these components, singing/production, music education and listening habits, overlap with the ones found by Cuddy et al. (2005). She identified a total of four components, the fourth being childhood memories. While Cuddy et al. (2005) and Wise (2009) were interested in the self-labeling as tone-deaf and consequently used it as outcome variable, we incorporated it as one of our components, 5 – self-assessment of musicality, into a multiple regression analysis with *d*^′^ as the outcome variable, because both studies found self-reports of tone-deafness not to be reliable and overlapping with the presence of congenital amusia. Our analysis showed that part of the variation in *d*^′^ scores can be accounted for by questionnaire information, more specifically music education and perception. The outcome of the meter subtest was also influenced by listening habits, a finding that is in agreement with Cuddy et al.’s (2005) findings. Contrary to Cuddy et al. (2005), we found no influence of the component music production on our outcome variable. We also found that only (and also only a small but significant amount of) the variation of scores on the memory subtest can be accounted for by one’s own assessment of musicality. It did not account for any other variability in *d*^′^ scores. Considering these results, it seems adequate to use at least a short questionnaire containing items about music education and music perception.

With our large-scale study we also tested the difference between MBEA scores that were obtained in a web-based experiment and those that were obtained under laboratory conditions with a computerized version of the MBEA. Participants scored significantly lower on all but the rhythm subtest if they were tested via the internet, probably due to uncontrollable external factors such as technical variance (e.g., internet speed or usage of headphones) and environmental factors (like noise or distractions; for an overview of drawbacks with web-based testing, see Reips, 2000; Dandurand et al., 2008). Our findings indicate that the results of studies employing web-based tests (such as Peretz et al., 2008; Provost, 2011) should be considered carefully, as they diagnose more congenital amusics (5.8 and 11.6%, respectively) than laboratory-based studies if the same cut-off scores are applied. A possible solution might be the application of different cut-off scores depending on the type of testing. However, it is questionable whether web-based testing of the MBEA should be used at all, since a controlled and quiet environment seems crucial for the success of perceptual and especially auditory research (cf. Krantz and Dalal, 2000).

In sum, we thus recommend the calculation of cut-off scores based on the SDT measures *d^′^* and *c* instead of percentage correct for all MBEA subtests separately (rather than averaging over subtests) and the additional use of a questionnaire and a further rhythmic subtest. We furthermore advise testing in the laboratory only. This way, a more reliable diagnosis of congenital amusia and a differentiation of amusic subtypes seem possible in the future.

## Conflict of Interest Statement

The authors declare that the research was conducted in the absence of any commercial or financial relationships that could be construed as a potential conflict of interest.
